# Defining Processing Times for Accelerator Produced ^225^Ac and Other Isotopes from Proton Irradiated Thorium

**DOI:** 10.3390/molecules24061095

**Published:** 2019-03-20

**Authors:** Jonathan Fitzsimmons, Justin Griswold, Dmitri Medvedev, Cathy S. Cutler, Leonard Mausner

**Affiliations:** 1 Isotope Production Laboratory, Collider-Accelerator Division, Brookhaven National Laboratory, Upton, NY 11973, USA; dmedvede@bnl.gov (D.M.); ccutler@bnl.gov (C.S.C.); lmausner@bnl.gov (L.M.); 2 Isotope and Fuel Cycle Technology Division, Oak Ridge National Laboratory, Oak Ridge, TN 37831, USA; griswoldjr@ornl.gov

**Keywords:** Isotope production, Actinium-225, Silver-111, MCNPX, Thorium, proton irradiation, fission products, Sr-82, critical process parameter

## Abstract

During the purification of radioisotopes, decay periods or time dependent purification steps may be required to achieve a certain level of radiopurity in the final product. Actinum-225 (Ac-225), Silver-111 (Ag-111), Astatine-211 (At-211), Ruthenium-105 (Ru-105), and Rhodium-105 (Rh-105) are produced in a high energy proton irradiated thorium target. Experimentally measured cross sections, along with MCNP6-generated cross sections, were used to determine the quantities of Ac-225, Ag-111, At-211, Ru-105, Rh-105, and other co-produced radioactive impurities produced in a proton irradiated thorium target at Brookhaven Linac Isotope Producer (BLIP). Ac-225 and Ag-111 can be produced with high radiopurity by the proton irradiation of a thorium target at BLIP.

## 1. Introduction

Typically, it is best to commence isotope purification as soon as possible after the End of Bombardment (EOB), to minimize losses due to decay. However, in some cases a cooling-off period is introduced to maximize the decay of shorter-lived radioimpurities and achieve the desired radionuclidic purity. To achieve the desired product quality, the United States Food & Drug Administration (FDA) requires that critical process parameters be identified, then monitored or controlled [1]. Both experimental and computational approaches can be used to determine optimal radiopurity, and establish optimal processing and irradiation times for a desired radioisotope. In this manuscript, the production of strontium-82 from a rubidium chloride target is used to illustrate the concept of optimal processing times and critical process parameters. The optimal processing times for Actinum-225 (Ac-225) from a thorium target, and subsequent fission products Silver-111 (Ag-111), Rhodium-105 (Rh-105), Astatine-211 (At-211), and Ruthenium-105 (Ru-105) produced in the thorium target are examined. It should be noted that two independent subcategories of radiopurity are discussed in this manuscript: radioisotopic purity and radionuclidic purity. Radionuclidic purity is defined as the absence of other radionuclides. Radioisotopic purity is defined as the absence of other radioisotopes of the same element [2].

Strontium-82 (Sr-82) is produced by the nuclear reaction Rubidium-85 (Rb-85) (p,4n) Sr-82, and Rb-87 (p,6n) Sr-82, and the isotope is purified using ion exchange chromatography [3,4,5,6]. The purified Sr-82 is used in a generator to produce Rb-82 for myocardial imaging [7]. Curie levels of Sr-82 have been produced in 14–21 days proton irradiations with a proton energy between 40–90 MeV. During the production of Sr-82, other strontium isotopes are coproduced, such as Sr-85 (T_1/2_= 64.849 days) and Sr-83 (T_1/2_= 32.41 h), which can impact the radioisotopic purity. Sr-85 decays to a stable Rb-85, which does not contribute to a radiation burden. Sr-83 decays to a radioactive Rb-83, which has a half-life of 86.2 days. The presence of Sr-83 complicates the purification of Sr-82, which is focused mainly on the separation of Sr-82 from stable and radioactive Rb isotopes. If the processing occurs too soon after the End of Bombardment (EOB), the Sr-83 would be present in the Sr-82 product and the Sr-83 would decay to Rb-83, contaminating the injectable Rb-82 product from the generator [7]. To eliminate this problem, it is critical that a separation of Sr-82 from Rb-83 is carried out at least ten days after the EOB. This allows the Sr-83 to decay away and not be present in the purified Sr-82, providing acceptable levels of radioisotopic purity.

The United States Department of Energy Isotope Program is producing Ac-225 from a high energy proton-irradiated thorium-232 target. In addition to various isotopes of actinium, the irradiation generates over 400 other isotopes, including fission products and other actinides. Some of the fission products have potential utility for imaging and therapy in nuclear medicine (Table 1). The purpose of this paper is to begin to evaluate the optimal processing times for the isotopes listed in Table 1, to achieve the highest possible radiopurity. 

## 2. Results

When possible, yields of relevant isotopes in this study were determined experimentally from previous irradiations, like those described in Griswold et al., 2016 [14]. In some cases, relevant isotopes were unable to be detected via gamma spectroscopy, either due to short half-lives or the absence of high intensity γ rays. MCNP6-generated cross sections were used to determine the radioactivity of relevant isotopes of ruthenium, rhodium, palladium, silver, astatine, and radon, produced during the 192 MeV proton irradiation of Th-232. 

### 2.1. Ac-225 Radiopurity

**Figure 1:**
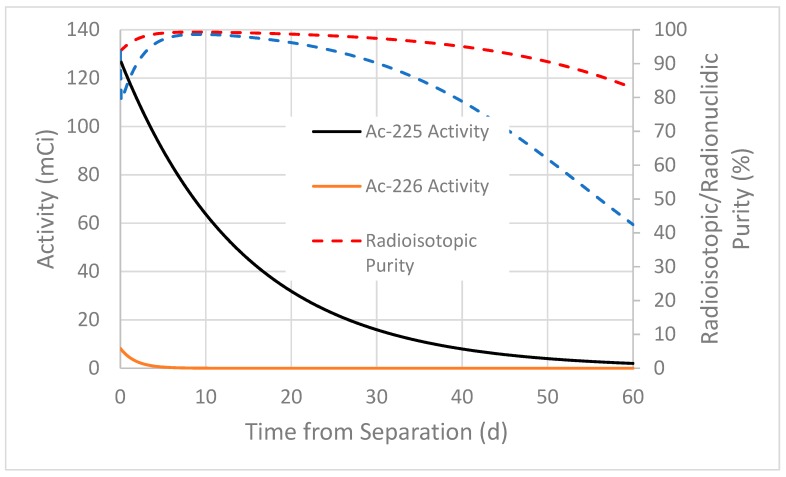
Experimentally calculated radionuclidic and radioisotopic purity of Actinum-225 (Ac-225) at BLIP. Radioisotopic purity includes only the Ac-225, Ac-226, and Ac-227 activity. Radionuclidic purity includes the activity of the previously mentioned actinium isotopes and the decay progeny generated by Ac-226 and Ac-227 over time. Irradiation parameters are specified in the caption for Table 2. Time 0 is the time corresponding to the end of processing (seven days after EOB).

**Figure 2:**
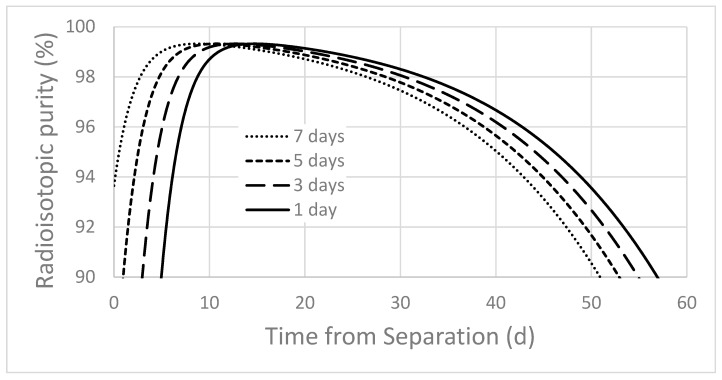
Radioisotopic purity of Ac-225 as a function of time from final chemical purification. The four curves depict the various durations between the EOB and the final purification. These values represent the radioisotopic purity of Ac-225, only considering all actinium isotopes (radioisotopic purity from Figure 1).

When Ac-225 is produced through a high energy proton bombardment of Th-232, significant quantities of Ac-226 (t_1/2_ = 29.37 h) and Ac-227 (t_1/2_ = 21.772 y) are coproduced. For a ten day irradiation of Th-232, at 192 MeV incident proton energy and 150 µA of current, approximately twice as much Ac-226 activity is generated as Ac-225 activity (Table 2). Decay from the coproduced Ra-225 (t_1/2_ = 14.9 d) is included in the yield shown in Table 2, and the method for determining the experimental effective cross section is shown elsewhere [14]. The radiopurity of Ac-225 from this process is highly dependent on irradiation time, and on the time from the End of Bombardment to the final actinium purification. As the time from the End of Bombardment to the final actinium purification is increased, the radioisotopic purity of Ac-225 increases due to the faster decay of Ac-226 compared to the decay of Ac-225; reaching a maximum value of 99.3% at about nine days. After nine days, the radioisotopic purity continually decreases, as the Ac-225 activity decreases relative to the longer-lived Ac-227 activity. Figure 1 shows the radioisotopic and radionuclidic purity of Ac-225 against time, for a seven day period between the EOB and the final purification. The effect of time between the EOB and the final purification is clearly shown in Figure 2.

### 2.2. Ag-111 Radiopurity

**Figure 3:**
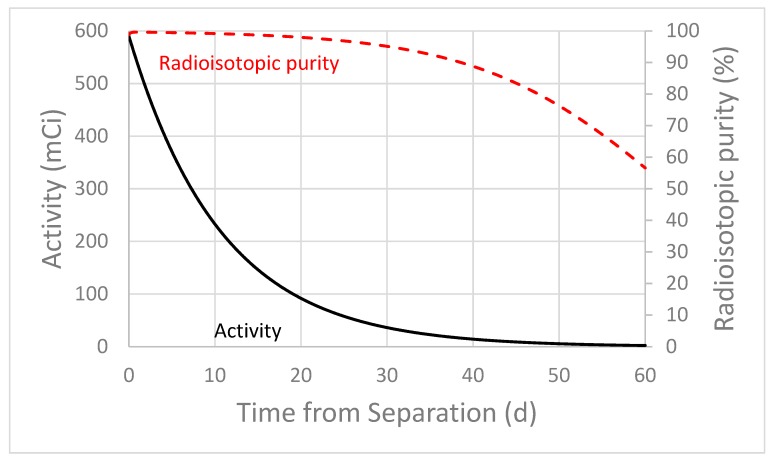
MCNP6 and experimentally calculated radioisotopic purity of Ag-111. Calculations assume silver purified from Ac-225 waste streams and irradiation parameters specified in the caption for Table 3.

Significant quantities of Ag-111 (t_1/2_ = 7.45 d) are also produced in the high energy proton irradiation of thorium. The total experimental yield of Ag-111 includes the decay of shorter-lived fission isobars such as Pd-111 (t_1/2_ = 23.4 min) and Rh-111 (t_1/2_ = 11 s). These isotopes will be in the process waste after Ac separation. As shown in Table 3, greater than 1 Ci each of Ag-111, Ag-112, Pd-112, and Ag-113 are produced in a ten days irradiation of a 1 g (450 mg cm^−2^) thorium target, at 192 MeV incident proton energy and 150 µA beam current. However, if a decay period is introduced between the EOB and final Ag purification, the radioisotopic purity of Ag-111 increases from 30% to >99% in seven days, due to the decay of the short-lived Ag isotopes. Radioisotopic purity is shown against time after a seven days processing period post-EOB in Figure 3.

### 2.3. Rh-105 Radionuclidic Purity

**Figure 4:**
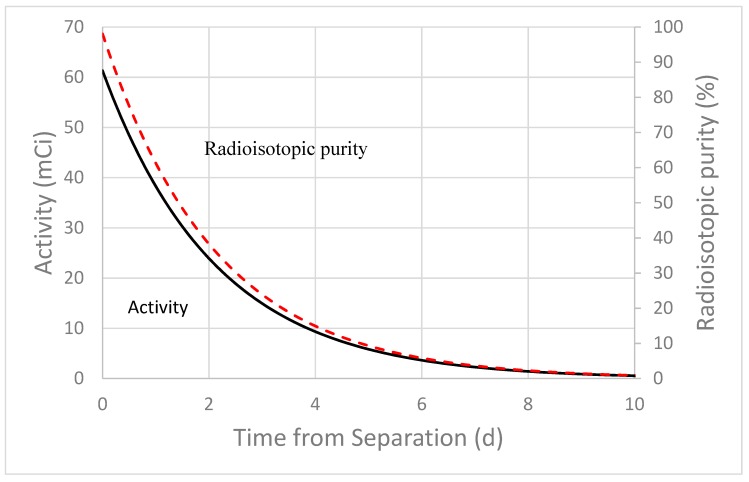
MCNP6 and experimentally calculated radioisotopic purity of Rh-105. Calculations assume rhodium purified from Ac-225 waste streams and irradiation parameters specified in the caption for Table 4.

Both Rh-105 (t_1/2_ = 35.36 h) and Ru-105 (t_1/2_ = 4.44 h) are coproduced in the high energy proton irradiation of thorium. Although Curie quantities of Ru-105 (this includes decay of shorter-lived fission products Tc-105 (t_1/2_ = 7.6 min) and Mo-105 (t_1/2_ = 35.6 s)) are generated as shown in Table 4, the short half-life limits the practicality of using Ru-105 produced through this method as a Rh-105 generator. Using the same irradiation parameters as mentioned for other isotopes above, Rh-105 with high radioisotopic purities can be achieved. The radioisotopic purity quickly declines with the Rh-105 half-life, as shown in Figure 4, due in part to the presence of the longer-lived Rh-99 and Rh-102.

### 2.4. At-211 Radiopurity

**Figure 5:**
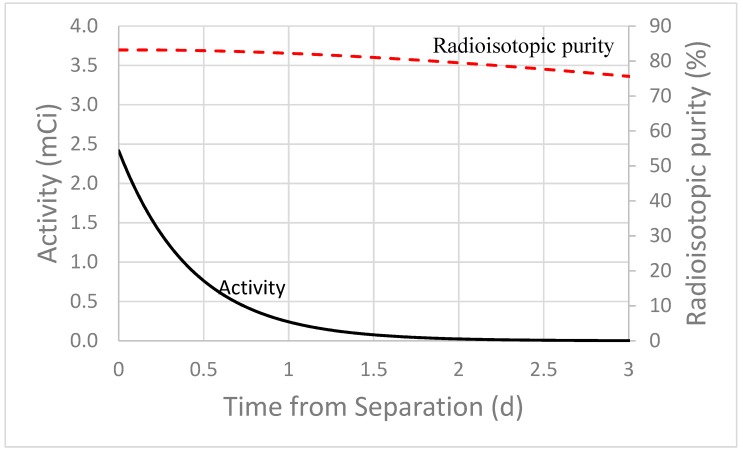
MCNP6 and experimentally calculated radioisotopic purity of At-211. Calculations assume astatine purified from Ac-225 waste streams and irradiation parameters specified in the caption for Table 5.

As shown in Table 5 and Figure 5, At-211 is also generated through the high energy proton irradiation of thorium. However, the seven days processing period employed for the other nuclides discussed in this paper is not appropriate for At-211 production due to its 7.21 h half-life. For the sake of discussion, the values generated in Table 5 and displayed in Figure 5 employ a one day processing period instead of a seven day processing period. Using the same irradiation parameters described for the other nuclides above but employing the one day decay period yields a total At-211 activity of less than 2.5 mCi, and a radioisotopic purity of ~85%. This includes contributions from the decay of Rn-211 (t_1/2_ = 14.6 h) to At-211.

## 3. Discussion

### 3.1. Considerations for the Processing Time for Ac-225

**Figure 6:**
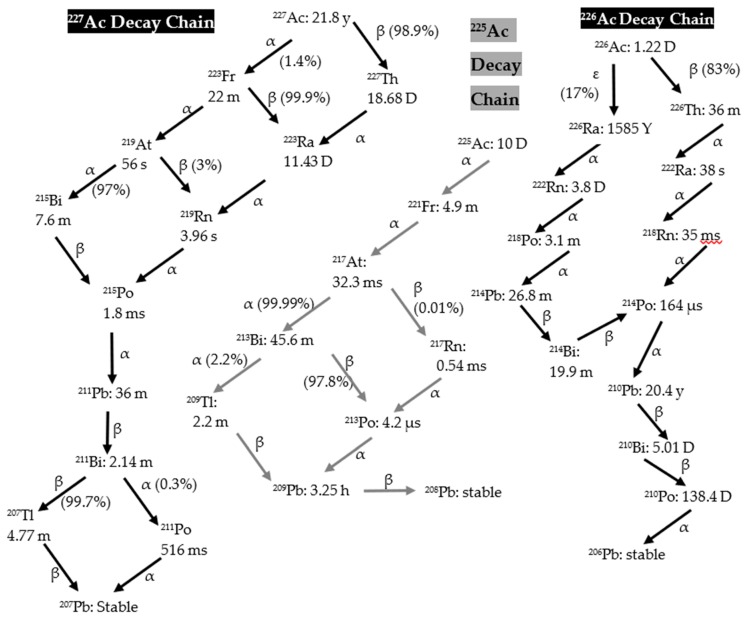
Ac-225, Ac-226, and Ac-227 decay chains [18].

Ac-225 (t_1/2_ = 10 d), Ac-226 (t_1/2_ = 29.37 h), and Ac-227 (t_1/2_ = 21.772 years) are coproduced during a proton irradiation of a Th target. Chromatography cannot separate Ac-226 and Ac-227 from Ac-225, so the optimal processing time becomes a critical parameter to produce the highest radioisotopic purity of Ac-225. Varying the incident proton energy upon the thorium target also impacts this ratio, but at lower incident proton energy the Ac-226/Ac-225 ratio worsens [14]. Note also that longer irradiations improve this ratio, as the Ac-226 production saturates after about five days, while Ac-225 levels continue to increase. The decay chains of Ac-225, Ac-226, and Ac-227 are illustrated in Figure 6. The Ac-226 decay chain has two major paths, one through the longer-lived Ra-226 (ε, 17%) and the other through Th-226 (β^−^, 83%). Due to the difference in half-lives, the ratio of Ac-226 to Ac-225 quickly decreases after the EOB. If actinium purification begins immediately after the EOB, the process could require an additional separation step to separate daughters of Ac-226 from the Ac-225 product, depending on the required radionuclidic purity. As seen in Table 2, the shorter half-life and higher cross section result in a higher amount of Ac-226 (433 mCi) produced during the irradiation compared to Ac-225 (207 mCi). Using the irradiation parameters mentioned in the above sections, Ac-225 produced at BLIP achieves 95% radionuclidic purity in three–four days after final actinium purification (Figure 1, Figure 2 and Table 2). Consider a scenario where processing starts shortly after the EOB to remove fission products and bulk thorium, using previously reported methods [15]. In such a case it is important to monitor the radionuclidic purity of the Ac-225 after final actinium purification as Ac-226 and Ac-227 decay daughter products grow in. An additional purification could be carried out after the initial purification, to increase the shelf-life by ensuring that Ac-226 and Ac-227 daughter products are separated. Alternatively, a cooling-off period can be used to reduce Ac-226 activity through decay, but this could result in a substantial amount of Ac-225 also lost to decay. Over time, the Ac-227 to Ac-225 ratio will increase in the purified Ac-225 product and will lead to a decrease in the Ac-225 radioisotopic purity. The end of processing and radiopurity should be evaluated for different beam energies and irradiation times. The DOE Isotope Program is preparing a Drug Master File (DMF) on accelerator produced Ac-225, to support future Ac-225/Bi-213 radiopharmaceutical Investigational New Drug (IND) applications to the FDA. 

### 3.2. Considerations for the Processing Time for Ag-111

Ag-111 obtained from a proton irradiated Th target would contain radioactive impurities of Ag with long half-lives (Ag-105 t_1/2_ = 41.29 d, Ag-110m t_1/2_ = 249.76 d) and short half-lives (Ag-112 t_1/2_ =3.13 h, Ag-113 t_1/2_ =5.37 h). The radioisotopic purity of Ag-111 is shown in Figure 3 and Table 3. To reduce impurities, purification should be completed after the EOB (Figure 3). Waiting for the decay of the impurities would result in an increase in radioisotopic purity, but also the loss of about half of the Ag-111 produced. Separation of Ag-111 from one of the waste streams of Ac-225 processing could be initiated, but introducing a separation step would be critical to remove ingrown daughters of short-lived Ag isotopes. Purification methods have been developed that could purify the Ag-111 from the Ac-225 waste streams [16]. Long-lived impurities (Ag-105, Ag-110m) could hinder the medical use of Ag-111 purified from Ac-225 waste streams.

### 3.3. Considerations for the Processing Times for Rh-105, Ru-105, and At-211

Direct purification of Rh-105 from the Ac-225 waste streams would result in >98% radioisotopic purity immediately after rhodium purification and decrease quickly with the half-life of Rh-105, as shown in Figure 4. Therefore, dedicated production approaches from the method described above for Rh-105 would result in acceptable radioisotopic purity for a short period. Direct purification of At-211 from the Ac-225 waste streams produced in the high energy proton irradiation of thorium is not practical due to its short half-life. Higher yields have been achieved through other production methods [17].

## 4. Materials and Methods

MCNP6 was used to calculate the theoretical cross sections of the radionuclides not previously detected in the proton irradiated thorium targets. The conditions used for the radiopurity calculations for Ac-225 at BLIP were: target density 450 mg/cm^2^, proton irradiation at 192 MeV, 150 µA for ten days, seven days shipping/processing time (except for At-211 as outlined above), cross sections for Ac-225, Ac-226, and Ac-227 were published previously [14]. All other experimental cross sections used in the calculations described in this work were determined in a similar method from previous irradiations but are currently unpublished.

## 5. Conclusions

Ac-225, Ag-111, and Rh-105 can be produced at high radionuclidic purity levels from a proton irradiated thorium target. Further studies may need to be performed to continue to optimize critical parameters such as irradiation and processing time, to deliver medical radionuclides with the highest purity possible from thorium targets irradiated with high energy protons.

## Figures and Tables

**Table 1:** Medical isotopes of interest coproduced during the proton irradiation of Thorium-232 (Th-232). Decay information from National Nuclear Data Center (nndc.bnl.gov).

Isotope	T_1/2_	Emission	Application	Ref.
**Ac-225**	10.0 d	5.830 MeV α	Alpha therapy, Generator for Bi-213	[8,9]
**Rh-105**	35.36 h	567.2 keV β, 318.9 keV γ	Radiotherapy	[10]
**Ru-105**	4.44 h	1193 keV β, 129 keV γ	Generator for Rh-105	[11]
**Ag-111**	7.45 d	1036.8 keV β, 342.13 keV γ	Radiotherapy	[12]
**At-211**	7.214 h	5.8695 MeV α	Alpha therapy	[13]

**Table 2:** Radioactive quantities of actinium isotopes produced in a proton irradiated thorium target produced at the Brookhaven Linac Isotope Producer (BLIP). Parameters for calculations: a 1 g Thorium-232 target (450 mg cm^−2^); proton irradiation at 192 MeV; 150 µA for ten days. Final separation is seven days post End of Bombardment (EOB).

Nuclide	T_1/2_	Activity (mCi)	
		EOB	Final Separation
Ac-225	10.0 d	206.91	127.37
Ac-226	29.37 h	433.01	8.21
Ac-227	21.772 y	0.42	0.42

**Table 3:** Radioactive quantities of silver and palladium isotopes produced in a proton irradiated thorium target produced at the BLIP. Parameters for calculations: a 1 g Thorium-232 target (450 mg cm^−2^); proton irradiation at 192 MeV; 150 µA for 10 days. Final separation is seven days post-EOB.

Nuclide	T_1/2_	Activity (mCi)	
		EOB	Final Separation
Ag-105	41.29 d	0.02	0.02
Ag-110m	249.76 d	2.04 ^a^	2.00 ^a^
Ag-111	7.45 d	1132.41 ^a^	590.41 ^a^
Ag-112	3.13 h	1407.91	4.73
Pd-112	21.04 h	1019.22	4.02
Ag-113	5.37 h	1239.17	0.00

^a^ Derived from experimentally measured cross sections.

**Table 4:** Radioactive quantities of ruthenium, rhodium, and palladium isotopes produced in a proton irradiated thorium target produced at the BLIP. Parameters for calculations: a 1 g Thorium-232 target; proton irradiation at 192 MeV; 150 µA for ten days. Final separation is seven days post-EOB.

Nuclide	T_1/2_	Activity (mCi)	
		EOB	Final Separation
Ru-97	2.83 d	17.08	3.08
Rh-99	16.1 d	0.93	0.69
Pd-100	3.63 d	0.60	0.16
Rh-100	20.8 h	0.80	0.21
Rh-101	3.3 y	0.01	0.01
Rh-102	207.3 d	0.31	0.31
Ru-103	39.247 d	218.52	193.11 ^a^
Ru-105	4.44 h	1265.42	0.00
Rh-105	35.36 h	1469.29	61.31
Ru-106	371.8 d	21.08	20.80 ^a^

^a^ Derived from experimentally measured cross sections.

**Table 5:** Radioactive quantities of various astatine and radon isotopes produced in a proton irradiated thorium target produced at the BLIP. Parameters for calculations: a 1 g Thorium-232 target; proton irradiation at 192 MeV; 150 µA for one day.

Nuclide	T_1/2_	Activity (mCi)	
		EOB	Final Separation
At-207	1.81 h	1.09	0.00
At-208	1.63 h	1.25	0.00
At-209	5.42 h	2.79	0.13
At-210	8.1 h	2.76	0.36
Rn-210	2.4 h	2.67	0.00
At-211	7.214 h	6.48	2.42
Rn-211	14.6 h	5.60	1.79
